# Editorial: Mentalization and Clinical Psychopathology

**DOI:** 10.3389/fpsyg.2022.837964

**Published:** 2022-02-17

**Authors:** Vanya L. Matanova, Drozdstoy S. Stoyanov, Olga Strizhitskaya

**Affiliations:** ^1^Department of Social, Organizational, Clinical and Pedagogical Psychology, Sofia University, Sofia, Bulgaria; ^2^Department of Psychiatry and Medical Psychology, Plovdiv Medical University, Plovdiv, Bulgaria; ^3^Department of Developmental and Differential Psychology, Saint Petersburg State University, Saint Petersburg, Russia

**Keywords:** mentalization, psychopathology, theory of mind, psychotherapy, coherence

Mentalization as a function and construct is defined as a specific form of imaginary mental activity related to the perception and interpretation of human behavior in terms of mental states motivated by intentions—e.g., needs, desires, feelings, beliefs, and goals (Fonagy and Target, 2006; Allen et al., 2008; Fonagy and Allison, 2013). Mentalization includes both cognitive and affective facets, or in other words, thinking about feelings and thinking about thinking (Fonagy and Target, 2006). This resource is developed in childhood through interpersonal interactions with a more mature mindset and it is based on the attachment quality with the main caregiver of the child. The dynamics of these two facets allows thinking about the quality of emotional reflection subjective experiences in childhood (Bateman and Fonagy, 2012). In the broadest sense, mentalization involves a process of transformation of elements via the Freudian concept called “binding,” or in English “binding.” It is an Ego function that transforms immediate physical quantities with associative mental ones in order to limit the free flow of arousal. It is a process of connecting ideas with each other, creating more sustainable forms, and establishing associative pathways, as part of secondary reprocessing, in order to adapt to external reality by creating sustainable representations of oneself and others.

In the last two decades, mentalization theory, mentalization-based therapy, and their relation to psychopathology have gained great popularity. This has opened new horizons for the definition and treatment of various psychopathological phenomena.

This Research Topic delivers special emphasis on whether and to what extent it is possible for mentalization to answer questions that have been raised by the diagnosis and treatment of various psychopathological conditions.

We assume that this provides a meaningful view of both the relevance of different aspects of mentalization and the introduction of new explanatory models for different types of psychopathology. Thus, the various papers contribute to the identification of psychopathological phenomena and their therapy.

In this Research Topic, we have attempted to consider integrating studies on various psychopathological phenomena. Particularly, nine articles have been published on various topics, they all are related to the analysis of mentalization, mentalization potential, the theory of mind, the theory of attachment, and their relations to various psychopathologies. Three of the articles present the difficulties and peculiarities of mentalization in therapeutic work (Wiwe; De la Cerda and Dagnino; Tohme and Kolev). One of the articles introduces a new approach to understanding the relations between attachment, reflexive parenting, mentalization, and body image (Bonev and Matanova). There is proposed in the latter an approach to the body image as a construct of human development, arising and developing from the relations of attachment, into the provision of security and protection. Another article introduces a new technology for assessing the condition during a clinical interview (Villanueva-Valle et al.).

Another focus is the article that discusses the coherence and influence of mentalization on burnout in health professionals in the COVID era (Stoyanova and Stoyanov). This paper is particularly relevant, given the challenges imposed by the ongoing epidemic situation on health care employees and the frequent manifestations of burnout among medical staff. Three articles are dedicated to the role of mental potential in the establishment of differential diagnostic criteria and biological markers for various psychopathological states (Helt et al.; Villanueva-Valle et al.; Vegni et al.).

A case study reports interesting perspectives on reflective parenting, mental potential, and theory of mind in children on the autistic spectrum disorders (Kostova).

The phenomenological content takes into account the modern understanding of mentalization and its relation with other mental constructs and psychological theories and provides new perspective for the understanding of mental disorders.

In conclusion, this is Research Topic addresses key issues in the relation between mentalization and psychopathology. In particular, it brings insight into the need for further investigation of the impact of mentalization and mental potential on major psychiatric conditions and outlines the contribution of mentalization to a wide range of psychopathological manifestations. This may support the formation of an evolutionary idea of how psychopathology could well be well explained and then re-attuned in terms of treatment by the theory of mentalization, theory of mind, and empathy.

Last but not least, we sincerely thank all the authors who provided their articles and actively contributed, allowing us to coordinate and edit this outstanding collection.

## Author Contributions

All authors listed have made a substantial, direct, and intellectual contribution to the work and approved it for publication.

## Conflict of Interest

The authors declare that the research was conducted in the absence of any commercial or financial relationships that could be construed as a potential conflict of interest.

## Publisher's Note

All claims expressed in this article are solely those of the authors and do not necessarily represent those of their affiliated organizations, or those of the publisher, the editors and the reviewers. Any product that may be evaluated in this article, or claim that may be made by its manufacturer, is not guaranteed or endorsed by the publisher.
